# Epidemiological analysis of respiratory viral etiology for influenza-like illness during 2010 in Zhuhai, China

**DOI:** 10.1186/1743-422X-10-143

**Published:** 2013-05-07

**Authors:** Hongxia Li, Quande Wei, Aijun Tan, Leyi Wang

**Affiliations:** 1 Microbiology Laboratory, Zhuhai Center for Disease Control and Prevention, Guangdong Province, 519000, China; 2 Division of Infectious Diseases, Cincinnati Children’s Hospital Medical Center, Cincinnati, OH, 45229, USA

**Keywords:** Influenza-like illness (ILI), Respiratory viral pathogens, Epidemiological analysis, Meteorology

## Abstract

**Background:**

Influenza-like illnesses (ILI), a subset of acute respiratory infections (ARI), are a significant source of morbidity and mortality worldwide. ILI can be caused by numerous pathogens, however; there is limited information on the etiology and epidemiology of ILI in China.

**Methods:**

We performed a one-year surveillance study (2010) of viral etiology causing ILI and investigated the influence of climate on outbreaks of ILI attributed to viruses at the Outpatient Department of Zhuhai Municipal People’s Hospital in Zhuhai, China.

**Results:**

Of the 337,272 outpatients who sought attention in the Outpatient Department of Zhuhai Municipal People’s Hospital in 2010, 3,747 (1.11%) presented with ILI. Of these patients presenting with ILI, 24.66% (924/3,747) had available samples and were enrolled in this study. At least one respiratory virus was identified in 411 patients (44.48%) and 42 (4.55%) were co-infected with two viruses. In patients co-infected with two viruses, respiratory syncytial virus (RSV) was detected in 50% (21/42). Among common viral pathogens detected, significant differences in age distributions were observed in seasonal influenza virus A (sFulA, H3N2) and B (sFluB), pandemic H1N1 2009 influenza viruses (H1N1pdm09), RSV, and adenovirus (ADV). Infections with sFluA (H3N2), sFluB, RSV, and human metapneumovirus (HMPV) had characteristic seasonal patterns. The incidences of sFluA (H3N2), ADV, and RSV correlated with air temperature. Alternatively, the incidence of sFluB correlated with relative air humidity.

**Conclusions:**

These results demonstrate that a wide range of respiratory viral pathogens are circulating in Zhuhai city. This information needs to be considered by clinicians when treating patients presenting with ILI.

## Introduction

Influenza-like illnesses (ILI), a subset of acute respiratory infections (ARI), are a leading cause of morbidity and mortality worldwide [1,2]. Of the 10 million deaths of children less than five years of age throughout the world, 1.9 million children died from ILI in the year 2000 [3]. Although the incidence of ILI is similar in both developed and developing countries, the mortality rates are higher in developing countries, such as China [4]. The elderly and individuals with compromised cardiac, pulmonary, or immune systems have the greatest risk of serious complications from ILI, which can be caused by any one of approximately 200 known viral species [5]. Among these viruses are well-recognized respiratory viral pathogens, including seasonal influenza virus A and B (sFluA and sFluB), parainfluenza virus (PIV), human metapneumovirus (HMPV), respiratory syncytial virus (RSV), and adenovirus (ADV). Patients infected by these diverse viral pathogens exhibit widely overlapping symptoms, rendering clinical diagnosis unreliable while severely limiting etiological and epidemiological studies.

Influenza viruses can cause annual recurrent epidemics affecting an estimated 5–15% of the population presenting with ARI worldwide. According to the World Health Organization (WHO), there are 3–5 million severe cases and 250,000–500,000 deaths globally due to influenza annually [6]. ADV are responsible for approximately 7–8% of reported childhood viral respiratory infections globally [7,8] and cause a broad spectrum of clinical disease, including respiratory tract infection, pharyngoconjunctival fever, conjunctivitis, hemorrhagic cystitis, and gastroenteritis. Although most infections are self-limited, ADV has been associated with severe and even fatal infections in both immunocompromised and healthy individuals [9-11]. In an immunocompromised host, ADV can cause severe localized disease or disseminated disease with multi-organ failure [12,13]. RSV can cause severe infections in infants and young children and is the leading cause of bronchiolitis in children under one year of age in the United States [14-17]. RSV outbreaks are responsible for a significant increase in hospital admissions during the winter season [15]. In 2005, at least 3.4 million cases of severe RSV-associated ARI requiring hospital admission occurred worldwide and between 66,000–199,000 children younger than five years of age died from RSV-associated ARI [18]. The signs and symptoms of HMPV infection are similar to those for RSV [19]. Since it was first described, HMPV has been reported in regions from around the world, with an incidence ranging from 3.9–43% [20-27]. PIV is an important cause of upper and lower respiratory tract illness, especially among children [3,28,29].

In temperate regions of the Northern Hemisphere such as the United States and Europe, the etiologic agents associated with ILI have been well characterized. However, in China, the epidemiology of viral etiology for ILI is poorly understood and there is only a paucity of data on whether climate factors affect disease incidence and prevalence. A laboratory-based influenza-like illness surveillance system is well established in Zhuhai city and has greatly contributed to the control of influenza virus infection due to advanced warning of outbreaks. Since 2004, seasonal influenza incidence rates have been monitored by detection of influenza viruses in nasopharyngeal swabs of patients presenting with ILI. The aim of the current study was to gain insight into the epidemiology of viral etiology responsible for ILI and whether any correlations exist between the incidences of these viral pathogens and meteorological parameters in Zhuhai city. PIV-1, PIV-2 PIV-3, RSV, HMPV, as well as sFluA (H1N1 and H3N2), sFluB, and H1N1pdm09 were detected by real-time reverse transcription polymerase chain reaction (real-time RT-PCR), and ADV was identified by real-time PCR in Zhuhai during 2010. This study provides clinicians with helpful information about the etiological patterns of patients presenting with complaints of acute respiratory symptoms.

## Results

### General findings

**Figure 1:**
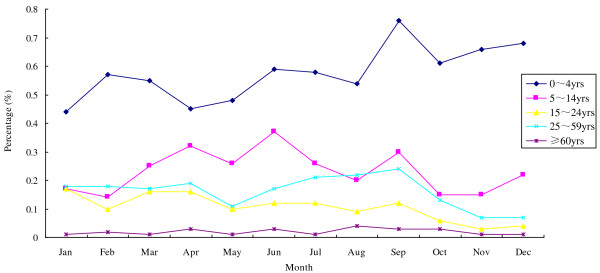
**The monthly distribution of ILI in outpatients by age group during 2010.** Of 337,272 outpatients who sought attention in the Outpatient Department of Zhuhai Municipal People’s Hospital, 3,747 (1.11%) presented with ILI in 2010. The prevalence of ILI (%) is shown for each month of 2010, divided into five different age categories.

Of 337,272 outpatients who sought attention in the Outpatient Department of Zhuhai Municipal People’s Hospital in 2010, 3,747 (1.11%) presented with ILI. The percentage of ILI in outpatients grouped by age during 2010 is shown in Figure 1. Of the patients presenting with ILI, 24.66% (924/3747) had available samples and were enrolled in this study. Of the 924 participants, 540 (58.44%) were male and 384 (41.56%) were female and ranged in age from one month to 78 years with a median age of four years and a mean age of 6.4 years (SD ± 8.7 years). Only two patients were older than 60 years of age, while 536 (58.01%) were under five years old and 308 (33.33%) were between the ages of 5–14.

### Proportion of respiratory viral pathogens

Of the 924 samples collected between January and December 2010, 411 (44.48%) were positive for at least one respiratory virus. A single infection was identified in 369 (39.94%) patients and co-infection with two viruses was observed in 42 (4.55%). The most frequently detected agent was sFluB (n = 90), followed by RSV (n = 66), ADV (n = 58), sFluA (H3N2) (n = 45), and HMPV (n = 43) in single infections. In the 42 specimens with co-infections, the most frequent combinations were sFluA (H3N2) plus RSV, followed by sFluA (H3N2) + PIV, sFluA (H3N2) + ADV, sFluB + RSV, PIV + RSV, PIV + ADV, RSV + HMPV, and RSV + ADV (Table 1).

**Table 1:** The proportion of respiratory viral pathogens in 924 patients with ILI

**Pathogens detected**	**Number**	**% of total no. of samples**	**% of positive samples**
**Single infection**	369	39.94	89.78
sFluA(H3N2)	45	4.87	10.95
H1N1pdm09	29	3.14	7.06
sFluB	90	9.74	21.9
PIV	38	4.11	9.25
RSV	66	7.14	16.06
MPV	43	4.65	10.46
ADV	58	6.28	14.11
**Coinfection**	42	4.55	10.22
sFluA(H3N2) + PIV	4	0.43	
sFluA(H3N2) + RSV	5	0.54	
sFluA(H3N2) + HMPV	2	0.22	
sFluA(H3N2) + ADV	4	0.43	
H1N1pdm09 + HMPV	1	0.11	
H1N1pdm09 + ADV	1	0.11	
sFluB + PIV	1	0.11	
sFluB + RSV	4	0.43	
sFluB + HMPV	1	0.11	
PIV + RSV	4	0.43	
PIV + HMPV	2	0.22	
PIV + ADV	4	0.43	
RSV + HMPV	4	0.43	
RSV + ADV	4	0.43	
HMPV + ADV	1	0.11	
**Total number of samples with pathogens**	411	44.48	
**No pathogen detected**	513	55.52	
**Total number of samples**	924		

### Gender distribution of virus infections

**Figure 2:**
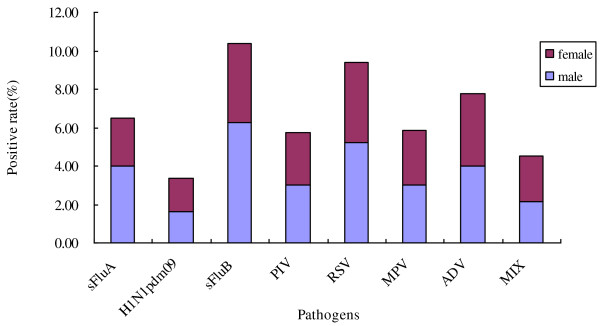
**Gender distribution of respiratory viral infection in 924 patients with ILI.** Of 924 patients with ILI, 540 (58.44%) were male and 384 (41.56%) female. Respiratory tract viruses were detected in 231 (46.80%) of the male patients and 181 (47.14%) of the female (*P* = 0.189, not significant). The age distribution is shown for each viral pathogen tested. Male = purple, female = blue.

There was no difference in the susceptibility to ILI based on gender in Zhuhai during 2010 (Figure 2). Of the 924 patients with ILI, 540 (58.44%) were male and 384 (41.56%) were female. Respiratory tract viruses were detected in 231 (46.80%) of the male patients and 181 (47.14%) of the female, an insignificant difference.

### Age distribution of virus infections

The distribution of viruses among different age groups is presented in Table 2. Viral infection was detected in 411 patients across all age groups except for the ≥ 60 year-old group. In children under five years of age, RSV was detected most frequently (13.06%), followed by sFluA (H3N2) (8.95%), which were significantly higher than other age groups (*P* < 0.001 and *P* = 0.009, respectively), followed by HMPV (6.72%) and ADV (6.72%). Meanwhile, patients between 5–14 years of age were mainly infected with sFluB (21.43%) and ADV (11.04%), which were also significantly higher than other age groups (*P* < 0.001 and *P =* 0.037, respectively). The highest incidence of H1N1pdm09 (10.91%) was in adults between 25–59 years of age, also significantly higher than other age groups (*P* = 0.033). Lastly, co-infection was more frequent in children under 5 years of age (5.04%).

**Table 2:** The distribution of viruses according to age group in 924 patients with ILI

	**<5 yr; n =536**		**5 yr-14 yr; n =308**		**15 yr-24 yr; n =23**		**25 yr-59 yr; n = 55**		**≥ 60yr; n = 2**		**Total; n = 924**
	Total no. (%)*	Coinfection no. (%)**	Total no. (%)*	Coinfection no. (%)**	Total no. (%)*	Coinfection no. (%)**	Total no. (%)*	Coinfection no. (%)**	Total no. (%)*	Coinfection no. (%)**	Total no. (%)*
sFluA(H3N2)	48(8.95)	12(2.24)	11(3.57)	3(0.97)	0(0.00)	0(0.00)	1(1.82)	0(0.00)	0(0.00)	0(0.00)	60(6.49)
H1N1pdm09	15(2.80)	1(0.19)	9(2.92)	1(0.32)	1(4.35)	0(0.00)	6(10.91)	0(0.00)	0(0.00)	0(0.00)	31(3.35)
sFluB	28(5.22)	2(0.37)	66(21.43)	4(1.30)	0(0.00)	0(0.00)	2(3.64)	0(0.00)	0(0.00)	0(0.00)	96(10.39)
PIV	34(6.34)	9(1.68)	17(5.52)	6(1.95)	1(4.35)	0(0.00)	1(1.82)	0(0.00)	0(0.00)	0(0.00)	53(5.74)
RSV	70(13.06)	13(2.43)	16(5.19)	8(2.60)	1(4.35)	0(0.00)	0(0.00)	0(0.00)	0(0.00)	0(0.00)	87(9.42)
HMPV	36(6.72)	7(1.31)	16(5.19)	4(1.30)	1(4.35)	0(0.00)	1(1.82)	0(0.00)	0(0.00)	0(0.00)	54(5.84)
ADV	36(6.72)	10(1.87)	34(11.04)	4(1.30)	2(8.70)	0(0.00)	0(0.00)	0(0.00)	0(0.00)	0(0.00)	72(7.79)
Positive samples	240(44.78)	27(5.04)	154(50.00)	15(4.87)	6(26.09)	0(0.00)	11(20.00)	0(0.00)	0(0.00)	0(0.00)	411(44.48)

Note. The age is given at the top of each column.

* Percentage of the total number of positive samples in the corresponding age group.

** Percentage of the total number of infections caused by this virus in the corresponding age group.

### Time distribution of respiratory tract viruses

**Figure 3:**
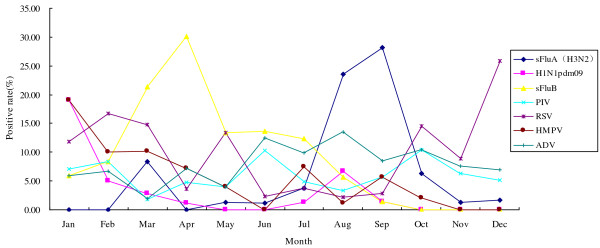
**Monthly distribution of respiratory viral infection in 924 patients with ILI.** Each viral pathogen tested in our study was plotted for each month in 2010. The percent of admitted patients infected is shown on the y axis as a function of the month on the x axis. In China, the four seasons were generally recognized as spring (March–May), summer (June–August), fall (September–November), and winter (December–February). Respiratory viral agents usually have characteristic seasonal patterns. The peak of sFluA (H3N2) was from August–September, sFluB from March–May, and the peak of H1N1pdm09 occurred in January. RSV infection occurred predominantly from December–February and usually was observed in co-infection with sFluA, sFluB, HMPV, and ADV. PIV and ADV were detected throughout the year without significant seasonality in 2010.

Respiratory viral pathogens usually have characteristic seasonal patterns. The peak of sFluA (H3N2) was from July–September, sFluB from March–May, while H1N1pbm09 and HMPV both peaked in January (Figure 3). RSV was another major viral pathogen of ILI and its infection presented predominantly from January–March and from October–December (Figure 3). On the other hand, PIV and ADV were detected throughout the year without significant seasonality during 2010 (Figure 3).

### Associations between each viral infection and meteorological parameters

**Figure 4:**
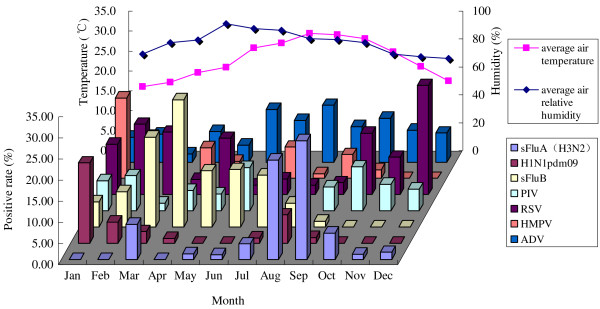
**The distribution of respiratory viral pathogens in the local climate of Zhuhai during 2010.** Zhuhai is located in the southern tip of Mainland China and is a subtropical coastal city with a humid climate and small diurnal air temperature range. The average monthly air temperature ranged 16–29.4°C and the average monthly relative humidity varied between 65.9-90.8% in Zhuhai during 2010. When the temperature rose, the number of patients infected with sFluA (H3N2) and ADV increased significantly (r = 0.592, *P* = 0.043 and r = 0.699, *P* = 0.011; respectively). Alternatively, the colder the temperature became, the number of patients infected with RSV increased (r = 0.699, *P* = 0.011). In addition, the prevalence of sFluB was significantly, positively correlated with air relative humidity (r = 0.697, *P* < 0.001).

The distribution of respiratory viral pathogens in Zhuhai city during 2010 correlated with the local climate (Figure 4, Table 3). When the temperature rose, the number of patients infected with sFluA (H3N2) and ADV increased significantly (r = 0.592, *P* = 0.043 and r = 0.699, *P* = 0.011; respectively). Alternatively, the colder the temperature became, the number of patients infected with RSV increased (r = 0.699, *P* = 0.011). In addition, the incidence of sFluB was increased significantly, positively correlated with air relative humidity (r = 0.697, *P* < 0.001).

**Table 3:** Correlations between each viral infection and meteorological parameters

	**sFluA(H3N2)**		**H1N1pdm09**		**sFluB**		**PIV**		**RSV**		**HMPV**		**ADV**	
	**r value**	***P *value**	**r value**	***P *value**	**r value**	***P *value**	**r value**	***P *value**	**r value**	***P *value**	**r value**	***P *value**	**r value**	***P *value**
Average temperature	0.592	0.043*	0.181	0.573	0.014	0.965	0.203	0.527	0.699	0.011*	0.352	0.262	0.699	0.011*
Average relative humidity	0.162	0.615	0.058	0.858	0.866	0.000*	0.413	0.183	0.476	0.118	0.204	0.524	0.042	0.897

*P* value is significant at 0.05 level(2-tailed).

## Discussion

During 2010, we conducted a one-year surveillance of viral etiology of ILI and found that of 337,272 outpatients who sought attention in the Outpatient Department of Zhuhai Municipal People’s Hospital, 3,747 (1.11%) presented with ILI. Of 3,747 outpatients with ILI, 924 (24.66%) had available samples and were enrolled in this study and ranged in age from one month to 78 years. Only two patients were older than 60 years of age, while 536 (58.01%) were under five years old and 308 (33.33%) were between the ages of 5–14. The age distribution of the 924 study participants was representative of the 3,747 patients presenting with ILI (Figure 1). This susceptibility of ILI in the younger age categories may be caused by several reasons. First, the immune system of children under five years old is naive and more susceptible of viral infections. Second, juveniles between the ages of 5–14 are generally students who are densely-gathered in schools promoting the spread of respiratory infectious. In addition, their immune systems are also still in development. Third, although aging impacts immunity to viral infection [30], patients older than 60 years of age usually have some comorbidities, such as hypertension, diabetes, or chronic bronchitis, and seek medical attention only when these primary diseases worsen when combined with ARI. Moreover, elderly people may self-administer according to their own experience. Of 924 samples, about 44.48% were positive for at least one virus, which is consistent with previous studies where viruses were detected in a range between 36% and 58% [31-34]. Among the tested viral pathogens, influenza virus, RSV, and HMPV exhibited characteristic seasonal patterns. In addition, there were correlations between the incidence of sFluA (H3N2), ADV, and RSV with air temperature, as well as between incidence of sFluB and air humidity. Therefore, the knowledge from our study is important for planning and implementation of effective interventions, including vaccinations.

During the study period, the introduction and emergence of H1N1pdm09 virus occurred, while sFluA (H1N1) was not detected in Zhuhai city. It is possible that the epidemic of H1N1pdm09 interfered with the appearance of sFluA (H1N1). The incidence of H1N1pdm09 was highest in adults between 25–59 years of age in 2010 (10.91%), with significant differences compared to other age groups (*P* = 0.033). Of the total cases of ILI, 10.39% were infected with sFluB, which was the predominant viral pathogen and occurred mainly from February–May in 2010, corresponding with a previous study of seasonal patterns [35]. Among 308 patients between 5–14 years of age, 66 (21.43%) were infected with sFluB and there was a significant difference in age distribution (*P* < 0.001). Infection with sFluA (H3N2) occurred predominantly in children less than five years old, which was significantly higher than other age groups (*P* = 0.009). In addition, the rate of sFluA (H3N2) + RSV was the most common viral co-infection in the respiratory tract of patients with ILI. In accordance with a previous study [36], the seasonal variability of sFluA (H3N2) was clear, presenting mainly from July–October, perhaps influenced by the epidemic of H1N1pdm09. We found that influenza strains were the most commonly detected viruses in agreement with other studies [37]. In addition, two significant correlations were observed; first, the higher the air temperature, the higher the incidence of sFluA (H3N2) (r = 0.592, *P* = 0.043), and second, the incidence of sFluB positively correlated with air relative humidity (r = 0.866, *P* < 0.001).

RSV, reported to be almost as common as influenza viruses, had the greatest impact on the youngest age groups [18,37] and was a major viral pathogen of ILI during the study period. Its incidence was 9.42% (87/924), which is consistent with the result reported by Fry *et al*. [38]. Our study showed that RSV infection occurred predominantly from January and March and October and December, with the majority of cases in children under five years old (*P* < 0.001). This is also in accordance with the study by Fry *et al.*[38], and RSV was the most commonly detected respiratory viral pathogens in co-infected patients. Additionally, RSV infection was negatively correlated with air temperature (r = 0.699, *P* = 0.011).

The detection rate for HMPV was 5.84% (54/924), which is consistent with the results of several other studies [20-23,25,27,39]. Although the study by van den Hoogen *et al.*[40] showed that most HMPV infections occur in children less than five years of age, our study did not show a significant difference in age distribution of HMPV infections. Previous studies indicate that the activity of HMPV in temperate climates peaks between December and February [24,41], where as in subtropics it peaks in the spring and summer months [42] and the peak of HMPV activity often coincides with or follows that of RSV [21,23]. In this study from Zhuhai city, located in subtropics, the incidence of HMPV peaked in January and did not follow that of RSV. HMPV infection did not correlate with air temperature unlike RSV, which is similar to another study [43] and was likely impacted by the circulation of H1N1pbm09 virus in Zhuhai city 2010.

Between two and seventeen percent of all cases of ARI are caused by PIV, making it second only to RSV as a cause of ARI among children under five years of age [44-46]. We detected PIV serotypes 1, 2, and 3 in 924 samples and the results showed that the incidence of all PIV serotypes combined was 5.74% (53/924). The lack of seasonality of PIV infection observed in this study is different from previous reports [45,47]. In addition, no significant differences were observed among the different age groups, possibly affected by the epidemic of H1N1pdm09 virus.

ADV is responsible for approximately 7%–8% of childhood viral respiratory infections worldwide [7,8], which is consistent with the result from the present study where the incidence of ADV infections was 7.79% (72/924) and ADV was mainly identified in patients under 15 years of age. Although our study determined that the activity of ADV was not seasonal, the infection rate of ADV appears to positively correlate with air temperature (r = 0.699, *P* = 0.011).

While the information provided by this study is valuable, there may be some limitations. First, recruitment bias could have affected the results of our study. However, Figure 1 shows that the age distribution of the 924 study participants with available samples was representative of the 3,747 patients presenting with ILI and sought attention at the Outpatient Department of Zhuhai Municipal People’s Hospital in Zhuhai city in 2010. Second, some viruses, such as rhinoviruses and coronaviruses, as well as bacteria, such as *M. pneumoniae* or *C. pneumoniae*, were not studied. The inclusion of these additional viral pathogens might greatly increase virus detection frequency, as indicated by a previous study [48] that reported a higher proportion of these viruses compared to influenza virus and might explain the relatively high proportion (55.52%, 513/924) of unidentified etiologies in our study.

In summary, the etiologic and epidemiologic data from the present study provide useful information to clinicians when treating patients presenting with ILI and to government officers when designing and implementing effective intervention plans.

## Methods

### Study population

The study population included every patient with ILI who sought attention in the Outpatient Department of Zhuhai Municipal People’s Hospital from January to December 2010. The Review Board of Zhuhai Medical Research Institute approved this study (Permit Number: 2009-41003). Written informed consent was obtained from all participants.

### Case definition

We used the following definition of ILI: “any person with a sudden onset of fever (≥38°C) and cough or sore throat fewer than three days in duration, and may be accompanied by general symptoms such as myalgia, prostration, headache, or malaise” [49].

### Clinical samples

Nasopharyngeal swabs were obtained from patients presenting with ILI. Samples collected in 3 ml of transport medium were transported immediately on ice in a container to the virology laboratory in Zhuhai Center for Disease Control and Prevention (CDC) and stored between 2–8°C. Separate aliquots of each clinical sample were prepared within 24 h and used for real-time PCR or RT-PCR analysis. A total of 15–20 samples were selected twice per week during the study period. Essential information (date of sample collection, patient’s initials, sex, age, clinical symptoms, and vaccination status) was collected for each specimen.

### Nucleic acid extraction

Commercially available kits (QIAamp Viral RNA/DNA Mini kit-QIAGEN, Germany) were used to extract viral RNA or DNA, according to the manufacturer’s instructions. The only modifications performed included the adjustment of the input volume to 180 μl and the elution volume 70 μl for all samples during both RNA and DNA extractions.

### Real-time RT-PCR or PCR

Real-time RT-PCR (sFluA, sFluB, H1N1pdm09, RSV, HMPV, and PIV) or real-time PCR (ADV) were used to detect the presence of virus in all samples. Commercially-available kits (Huayin Biology Company, China) were used to identify sFluA, sFluB, and H1N1pdm09. The oligonucleotide sequences [50-52] used to detect RSV, PIV, ADV, and HMPV are displayed in Table 4. The reactions were set up in a total volume of 25 μl containing 5 μl of genomic RNA or DNA template and 20 μl of universal master mix (TAKARA one-step PCR kit (AML)). Real-time RT-PCR was performed on the ABI 7500 fast sequence detection system and reaction conditions were designed as follows: RT at 50°C for 30 min, an initial denaturation at 95°C for 10 min, followed by 40 cycles of denaturation (95°C for 15 s) and annealing and extension (60°C for 40 s).

**Table 4:** Sequence details of all primer-probe combinations

**Virus type**	**Target**	**Amplicon**	**Oligonuleotide sequence(5′-3′)**	**Nucleotide positions**	**GenBank accession no.**
		**length(bp)**	**Forward**		
			**Reverse**		
			**Probe**		
Parainfluenza 1	HN gene	109	GTTGTCAATGTCTTAATTCGTATCAATAATT	1191–1220	U70948
			GTAGCCTACCTTCGGCACCTAA	1278–1299	
			TTGGAATAGTCTCGACAACAATCTTTGGCCTA	1232–1263	
Parainfluenza 2	HN gene	90	GCATTTCCAATCTTCAGGACTATGA	767–791	D00865
			ACCTCCTGGTATAGCAGTGACTGAAC	831–856	
			CCATTTACCTAAGTGATGGAATCAATCGCAAA	795–826	
Parainfluenza 3	HN gene	136	AGTCATGTTCTCTAGCACTCCTAAATACA	779–807	L25350
			ATTGAGCCATCATAATTGACAATATCAA	887–914	
			AACTCCCAAAGTTGATGAAAGATCAGATTATGCA	828–861	
Respiratory syncytial virus	F gene	90	AACAGATGTAAGCAGCTCCGTTATC	789-813	AF067125
			CGATTTTTATTGGATGCTGTACATTT	853–878	
			TGCCATAGCATGACACAATGGCTCCT	822-847	
Human metapneumovirus	N gene	163	CATATAAGCATGCTATATTAAAAGAGTCTC	89-116	AB503857
			CCTATTTCTGCAGCATATTTGTAATCAG	224–251	
			TGCAATGATGAGGGTGTCACTGCGGTTG	149-176	
			TGTAATGATGAGGGTGTCACTGCGGTTG		
Adenoviru A	Hexon gene	135	GGTCTGGTGCAATTCGCC	17818–17835	X73487
			CACGGGCACAAAACGCA	17936–17952	
			CCACGGACACCTACTTCACCCTGGG	17840–17864	
Adenoviru B	Hexon gene	138	CGCCGGACAGGATGCTT	45–61	X76549
			CTACGGTCGGTGGTCAC	166–182	
			AGTCCGGGTCTGGTGCAGTTCGCC	73–96	
Adenoviru C	Hexon gene	143	ACCTGGGCCAAAACCTTCTC	2884–2903	J01966
			CGTCCATGGGATCCACCTC	2940–2958	
			AACTCCGCCCACGCGCTAGA	2910–2929	
Adenoviru D	VA gene	75	AAAAACGAAAGCGGTTGAGC	2–21	U10675
			CGGGTCGAGACGGGAGT	128–144	
			CCAATACCACGTTAGTCGCGGCT	104–126	
Adenoviru E	Hexon gene	113	CAACACCTACTCGTACAAAGTGCG	225–248	X84646
			TAGGTGCTGGCCATGTCCA	281–299	
			CGCCCACGGCCAGCGTGT	251–268	
Adenoviru F	1.5 χ-2 gene		CCCGTGTTTGACAACGAAGG	31277–31296	L19443
			TTAGAGCTAGGCATAAATTCTACAGCA	31363–31389	
			ATCGACAAGGACAGTCTGCCAACACTAACG	31326–31355	

The sensitivity of the methods used in this study matched that of the US CDC international Pneumonia Surveillance Project in 2009, where the Zhuhai CDC and Zhuhai Municipal People’s Hospital participated was one of the China sites. The virology laboratory at the Zhuhai CDC was responsible for identifying respiratory viral pathogens.

### Climate data

The Zhuhai Bureau of meteorology supplied daily values for average air temperature and relative humidity during the period from 1 January – 31 December 2010.

### Statistical methods

The epidemiological forms were entered into a database created in Microsoft (MS) Office Access 2003, and data were analyzed using dynamic tables in MS Excel 2003. Bivariate correlation used to analyze associations between each viral infection and meteorological parameters and the Chi-square test used to compare between-group differences in percentages were performed with SPSS software version 11.5 (SPSS Inc. Chicago, IL, USA). *P* values less than 0.05 (*P* < 0.05) were considered significant.

## Abbreviations

ILI: Influenza-like illnesses; ARI: Acute respiratory infections; sFluA and sFluB: Seasonal influenza virus A and B; PIV: Parainfluenza virus; HMPV: Human metapneumovirus, RSV, Respiratory syncytial virus; ADV: Adenovirus

## Competing interests

The authors declare that they have no competing interests.

## Authors’ contributions

HL, QW, AT, LW conceived the study. HL helped in data acquisition and prepared the first draft of the paper. LW assisted in revising drafts of the manuscript. All authors read and approved the final manuscript.
